# An automated algorithm for quantitative morphometry of thoracic and lumbar vertebral bodies in lateral radiographs

**DOI:** 10.1093/jbmrpl/ziaf017

**Published:** 2025-01-25

**Authors:** Shoutaro Arakawa, Akira Shinohara, Daigo Arimura, Takeshi Fukuda, Yukihiro Takumi, Kazuyoshi Nishino, Mitsuru Saito

**Affiliations:** Department of Orthopaedic Surgery, The Jikei University School of Medicine, Tokyo 105-8461, Japan; Department of Orthopaedic Surgery, The Jikei University School of Medicine, Tokyo 105-8461, Japan; Department of Orthopaedic Surgery, The Jikei University School of Medicine, Tokyo 105-8461, Japan; Department of Radiology, The Jikei University School of Medicine, Tokyo 105-8461, Japan; Medical Systems Division, Shimadzu Corporation, Kyoto 604-8511, Japan; Medical Systems Division, Shimadzu Corporation, Kyoto 604-8511, Japan; Department of Orthopaedic Surgery, The Jikei University School of Medicine, Tokyo 105-8461, Japan

**Keywords:** osteoporosis, radiology, bone morphometry, vertebral fractures, fracture prevention, fracture risk assessment

## Abstract

This exploratory study developed and evaluated an artificial intelligence (AI)–based algorithm for quantitative morphometry to assess vertebral body deformities indicative of fractures. To achieve this, 709 radiographs from 355 cases were utilized for algorithm development and performance evaluation. The proposed algorithm integrates a first-stage AI model to identify the positions of thoracic and lumber vertebral bodies in lateral radiographs and a second-stage AI model to annotate 6 landmarks for calculating vertebral body height ratios (*C/A*, *C/P*, and *A/P*). The first-stage AI model achieved a sensitivity of 97.6%, a precision of 95.1%, and an average false-positive ratio of 0.43 per image for vertebral body detection. In the second stage, the algorithm’s performance was evaluated using an independent dataset of vertebrae annotated by 2 spine surgeons and 1 radiologist. The average landmark errors ranged from 2.9% to 3.3% on the X-axis and 2.9% to 4.0% on the Y-axis, with errors increasing in more severely collapsed vertebrae, particularly at central landmarks. Spearman’s correlation coefficients were 0.519-0.589 for *C/A*, 0.558-0.647 for *C/P*, and 0.735-0.770 for *A/P*, comparable with correlations observed among human evaluators. Bland–Altman analysis revealed systematic bias in some cases, indicating that the algorithm underestimated anterior and central height collapse in deformed vertebrae. However, the mean differences and limits of agreement between the algorithm and external evaluators were similar to those among the evaluators. Additionally, the algorithm processed each image within 10 s. These findings suggest that the algorithm performs comparably with human evaluators, demonstrating sufficient accuracy for clinical use. The proposed approach has the potential to enhance patient care by being widely adopted in clinical settings.

## Graphical Abstract

**Graphical Abstract:**
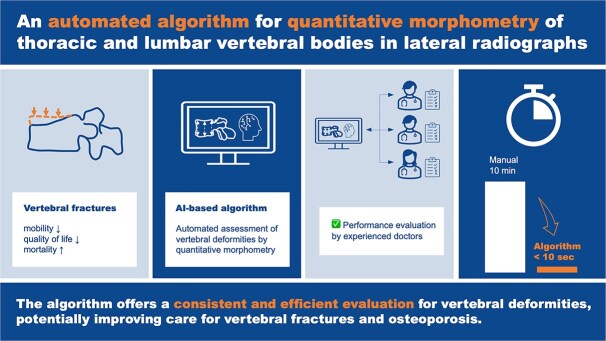

## Introduction

Vertebral fractures predominantly occur in older adults and individuals with osteoporosis, significantly impairing health by causing increased pain, restricted mobility, subsequent fractures, and a higher mortality rate, with a 5-yr survival rate below 30%.^1^ Additionally, the global prevalence of osteoporosis is 19.7%.^2^ The risk of vertebral fractures is further elevated by lifestyle-related diseases such as diabetes, chronic kidney disease, chronic obstructive pulmonary disease (COPD), dyslipidemia, and prolonged steroid use.^3^ Moreover, two-thirds of vertebral fractures are asymptomatic,^4^^,^^5^ emphasizing the necessity of effective detection methods, particularly as the demand for diagnostic capabilities continues to grow.

Currently, there is no established gold standard for the radiological diagnosis of vertebral fractures. Instead, comprehensive diagnoses rely on a combination of clinical examinations and imaging modalities such as CT and MRI. Lateral DXA images are commonly employed for fracture screening due to their low radiation exposure and simultaneous capability to measure bone mineral density.^6^ However, the low resolution of DXA images limit their utility for analyzing the thoracic spine and detecting subtle fractures.

Quantitative morphometry (QM), introduced in the 1960s, quantitatively evaluate vertebral deformities indicative of fractures.^7–14^ This method involves annotating 6 landmarks on the upper and lower edges of the anterior, central, and posterior regions of each vertebral body in lateral radiographs. Using these landmarks, anterior edge height (*A*), central height (*C*), and posterior edge height (*P*) are measured to calculate vertebral body height ratio: *C/A*, *C/P*, and *A/P*. While QM provides objective and precise measurements, its complexity and time-consuming nature have hindered its widespread clinical adoption. To address these challenges, Genant et al.^15^ proposed a semiquantitative (SQ) method in 1993. This approach visually categorizes the degree of vertebral body height and cross-sectional area reduction into 4 levels, eliminating the need for direct measurements. Although simpler and widely used in clinical practice and research, the SQ method has limitations, including evaluator subjectivity,^16^ difficulty in standardization, susceptibility to over- or underestimation,^17^ limited utility for follow-up evaluations, and the need for specialized training to ensure consistency.^18^

In recent years, artificial intelligence (AI)-based methods for vertebral fracture evaluation have gained attention. Several AI systems have been developed to detect vertebral fractures in the lumbar lateral radiographs^19^^,^^20^ or to quantitatively measure vertebral body and intervertebral disc heights using MRI.^21^ A notable example is the deep learning-based system developed by Suri et al.^22^ which automatically measures the vertebral body heights from radiographs, CT, and MRI images. However, this system is restricted to specific spinal regions (T10 to L5) and does not include mid-thoracic vertebrae, where fractures frequently occur,^23^ particularly in patients with respiratory conditions such as COPD^24^^,^^25^ or, more recently, COVID-19-related complications.^26^ These limitations of prior research highlight the pressing need for a clinically applicable system capable of efficiently and comprehensively evaluating vertebral body deformity indicative of fractures across the thoracic and LS.

To address these limitations, we developed a novel AI-based algorithm for QM of vertebral bodies using thoracic and lumbar radiographs. This exploratory 2-stage, deep learning-based algorithm automatically identifies the position of each vertebral body (first-stage AI) and calculates vertebral body height ratios *C/A*, *C/P*, and *A/P* (second-stage AI) within seconds, significantly reducing analysis time. We evaluated the algorithm’s performance by comparing its measurements with those of external evaluators, demonstrating its potential to enhance vertebral deformity assessments in clinical practice.

## Materials and methods

### Data collection

**Figure 1:**
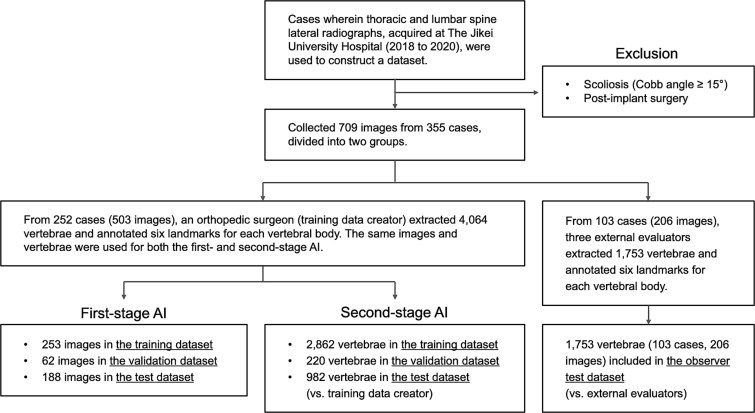
Flowchart of the data collection.

Data were retrospectively collected from patient records at The Jikei University Hospital between January 1, 2018, and October 31, 2020. Patient records that included frontal and lateral radiographs of the thoracic and LS were identified and reviewed. Patients who had undergone spinal fusion or presented with scoliosis characterized by a Cobb angle ≥15° were excluded. The final dataset consisted of 709 radiographic images (354 thoracic and 355 lumbar) from a total of 355 cases. Participants had an average age of 62.9 yr, with a gender distribution of 41% male and 59% female. Nearly all participants were of Japanese ethnicity. The dataset was subsequently divided into 2 groups (Figure 1).

For the training and test datasets group, an experienced orthopedic surgeon with over 10 yr of clinical practice (training data creator) annotated 6 landmarks on each of the 4064 vertebral bodies identified in 503 images corresponding to 252 cases. These vertebrae ranged from the fourth thoracic (T4) to the fifth lumbar (L5). Landmarks annotation followed the method proposed by Genant et al.,^15^ with landmarks placed at the center of the vertebral body edges in cases where the left and right sides were misaligned. The same set of 503 images and 4064 vertebrae was used for both the first- and second-stage AI models. For the first-stage AI, the data were randomly divided into a training dataset (253 images), a validation dataset (62 images), and a test dataset (188 images). For the second-stage AI, the data were randomly split into 2862 vertebrae for training, 220 vertebrae for validation, and 982 vertebrae for testing.

For the observer test dataset group, 3 external evaluators—2 spine surgeons and 1 radiologist, each with over 10 yr of clinical experience—annotated 6 landmarks on 1753 vertebral bodies from 206 images corresponding to 103 cases. To ensure consistency, the training data creator provided 15-20 annotated vertebrae as examples, and all evaluators adhered to the landmark annotation methodology outlined by Genant et al.^15^ Importantly, the 103 cases in the observer test dataset group were entirely independent of those in the training and test datasets.

This study adhered to the ethical standards set forth in the Helsinki Declaration and was approved by the Ethical Committee for Clinical Research at The Jikei University School of Medicine (Approval No. 31-078[9577]).

### Algorithm development

**Figure 2:**
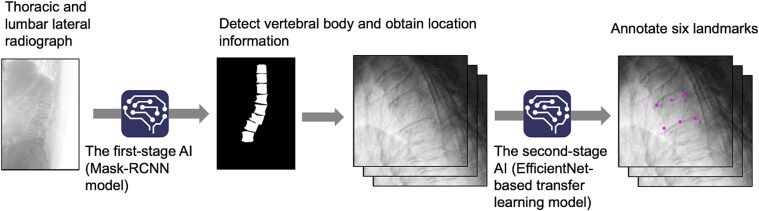
Flowchart of the proposed algorithm.

To automate the determination of landmarks, we developed an algorithm utilizing 2 AI models: a Mask Region Convolutional Neural Network (Mask R-CNN) and an EfficientNet-based transfer learning model with a modified head section. The first stage employs Mask R-CNN to identify the position of each vertebral body in lateral radiographs of the thoracic and LS. The second stage utilizes the EfficientNet-based model to determine 6 landmarks for each vertebral body detected in the first stage, enabling the calculation of vertebral body height ratios (Figure 2).

Mask R-CNN, a convolutional neural network (CNN), is a framework for instance segmentation that efficiently detects target objects in images while generating high-accuracy segmentation masks for each instance.^27^ In this algorithm, the Mask R-CNN produces segmentation images of individual vertebral bodies from lateral radiographs. The center of each vertebral body is calculated by determining the center of gravity coordinates from the segmentation results through a post-processing program. The training dataset for the first stage was created by annotating vertebral body landmarks, which was then used to manually draw contours along the vertebral body edges. To improve positional detection performance, the post-processing step excluded false detections by applying the Random Sample Consensus (RANSAC) algorithm^28^ to fit a polynomial curve (up to the fourth degree) to the spinal curve using all detected centers. Vertebral body centers deviating significantly from this curve were discarded. The refined center positions were then used to crop individual vertebral body image (224 × 224 pixels) from the radiograph. Only these cropped images served as inputs for the second-stage AI model, without segmentation data from the first stage.

The second-stage AI model is based on EfficientNet, a deep learning framework that achieve high image recognition accuracy by sequentially scaling up the width, depth, and resolution of the CNN model using a fixed ratio.^29^ Among EfficientNet models (B0-B7), the EfficientNetB2 model was selected as the base for this algorithm. The head section, which provides the final output, was modified to directly generate the coordinates of the 6 landmarks for each vertebral body image. Transfer learning was employed to fine-tune the model’s feature extraction capabilities, adapting it to landmark detection tasks.^26^ A post-processing program was developed to calculate the Euclidean distances between the anterior (*A*), central (*C*), and posterior (*P*) edges using the 6 landmark coordinates output by the second-stage AI model. These distances were then used to compute vertebral body height ratios. The 2 AI models demonstrated high measurement accuracy across diverse clinical images, accounting for variability in patient anatomy and positioning. This robustness was achieved through image augmentation, which increased the diversity of the training data using random preprocessing techniques, including edge enhancement, noise addition, resizing, contrast adjustment, and rotation.

### Performance evaluation

#### First-stage AI

The performance of the first-stage AI, vertebral body detection was evaluated using the test dataset comprising188 images (Figure 1). A total of 1582 vertebral bodies identified by the training data creator served as the reference standard. The AI’s predictions were categorized as follows:

- **True Positive (TP):** AI detections matching the reference standard.
- **False Positive (FP):** AI detections not present in the reference standard.

Since explicit non-vertebral regions were not annotated, True Negative (TN) and False Negative (FN) metrics were not calculated. The performance metrics assessed included the following:

- **Sensitivity:** TP/Reference Standard.
- **Precision:** TP/(TP + FP).
- **False Positive Rate per Image** = FP/Total Images.

#### Second-stage AI

The performance of the second-stage AI was assessed by evaluating landmark error and the correlation and agreement of vertebral body height ratios calculated by the algorithm. Landmark errors were separately evaluated along the X and Y axes, adjusted for each vertebral body’s width and height to account for size variations across images. Errors were expressed as percentages relative to the vertebral body’s dimensions. Correlation was determined through correlation analysis, while agreement between algorithm-generated and reference measurements was evaluated using Bland–Altman analysis. The evaluation was conducted using 2 datasets.

- Test Dataset consisted of 982 vertebral bodies annotated by the training data creator.
- Observer Test Dataset consisted of 1753 vertebral bodies from 206 images annotated by 3 external evaluators.

Subgroup analysis was performed to evaluate the algorithm’s performance across different levels of vertebral collapse. Collapse was categorized based on QM status as <20%, 20%-25%, 25%-40%, and >40%.^22^

No manual adjustments were made to the landmarks after the algorithm’s automated calculations.

#### Measurement time

The measurement time for total throughput of the first and second stage was assessed using 10 randomly selected images from the test dataset, including thoracic and LS radiographs. The average processing time was calculated on a standard Windows 10 laptop (11th Gen Intel Core i5-1135G7 CPU @ 2.40 GHz, 8 GB RAM) without a dedicated GPU.

### Statistical analysis

The normality of data distributions was evaluated using the Shapiro–Wilk test. Correlations between parameters were determined using Pearson or Spearman correlation tests, depending on the normality of the data. Bland–Altman analysis was used to assess agreement, with limits of agreement (LOA) calculated as the mean difference ±1.96 SDs derived from the observed measurement differences. The LOA represented the range within which 95% of the differences between the 2 measurement methods were expected to fall. Statistical significance was defined as *p* < .05. All statistical analyses were performed using the SAS Studio version 3.81 (SAS Institute Inc.).

## Results

### Verification of the first-stage AI

The first-stage AI, incorporating post-processing for center position correction, successfully detected 1544 of the 1582 reference vertebral bodies (true positives, TP) and identified 79 false positives (FP) across 188 radiographs. The performance metrics were as follows:

- Sensitivity: 97.6%.
- Precision: 95.1%.
- False Positive Rate per Image: 0.43.

### Verification of the second-stage AI using the test dataset

The overall landmark error for the test dataset was 2.1% on the X-axis and 2.3% on the Y-axis. Thoracic images exhibited slightly higher relative errors (2.2% and 2.4%, respectively) compared with 2.0% and 2.2% for lumbar images. Landmark error varied with to the degree of vertebral collapse, reaching up to 2.5% for collapse levels of <25%, with greater variability observed on the Y-axis for higher levels of collapse. This variability was particularly pronounced in anterior-upper, anterior-lower, and central-lower landmarks.

Based on the correlation analysis, Spearman’s correlation coefficient (*r_s_*) was 0.605 for *C/A*, 0.721 for *C/P*, and 0.798 for *A/P***.** All correlations were statistically significant (*p* < .0001).

**Figure 3:**
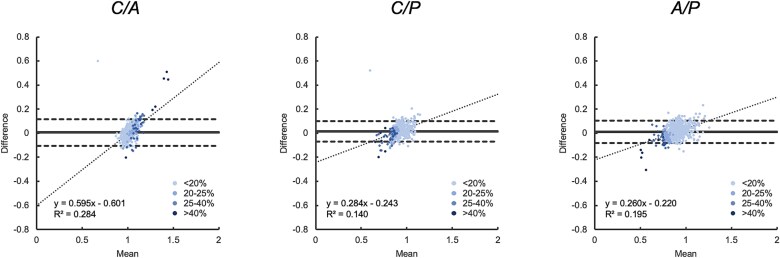
Agreement of calculated vertebral body height ratios in the test dataset (algorithm versus training data creator) displayed as a Bland–Altman plot. The solid line represents the mean difference, and the dashed line indicates the LOA (mean difference ± 1.96 SD). The data are shaded and classified according to the degree of vertebral body collapse determined by the training data creator. MD and LOA for each ratio are as follows: *C/A*: MD = 0.006, LOA = [−0.104, 0.117]; *C/P*: MD = 0.016, LOA = [−0.070, 0.101]; *A/P*: MD = 0.012, LOA = [−0.082, 0.106]. A moderate positive linear trend is observed in the Bland-Altman plot for the *C/A* ratio (slope = 0.595, *p* < .0001, *R*^2^ = 0.284). While significant linearity was also noted for *C/P* and *A/P* ratios, the explanatory power was lower. *n* = 982. Abbreviations: LOA, limits of agreement; MD, mean difference; *R*^2^, *R*-squared.

Additionally, agreement was examined using Bland–Altman analysis (Figure 3), which revealed a moderate positive linear trend for the *C/A* ratio (slope = 0.595, *p* < .0001, *R*^2^ = 0.284). Post hoc analysis indicated that the proportion of vertebral bodies within the LOA decreased with increasing levels of collapse (Table 1).

**Table 1:** Bland–Altman analysis of the test dataset. As vertebral collapse progresses according to QM status, the MD deviates further from 0, the SD increases, and the proportion of data points within the LOA decreases for each vertebral body height ratio.

	**QM status (%)**	***n***	**MD**	**LOA**	**Within LOA (%)**
***C/A***	<20	886	0.000 ± 0.046	−0.104 to 0.117	98.6
	20-25	57	0.029 ± 0.055		80.2
	25-40	33	0.040 ± 0.090		69.7
	>40	6	0.268 ± 0.242		0.0
***C/P***	<20	886	−0.001 ± 0.041	−0.070 to 0.101	98.4
	20-25	57	−0.011 ± 0.040		92.7
	25-40	33	−0.027 ± 0.049		90.9
	>40	6	−0.074 ± 0.083		66.7
***A/P***	<20	886	0.001 ± 0.043	−0.082 to 0.106	97.7
	20-25	57	−0.032 ± 0.028		92.7
	25-40	33	−0.053 ± 0.051		90.9
	>40	6	−0.155 ± 0.086		33.3

Abbreviations: LOA, limit of agreement; MD, mean difference.

**Figure 4:**
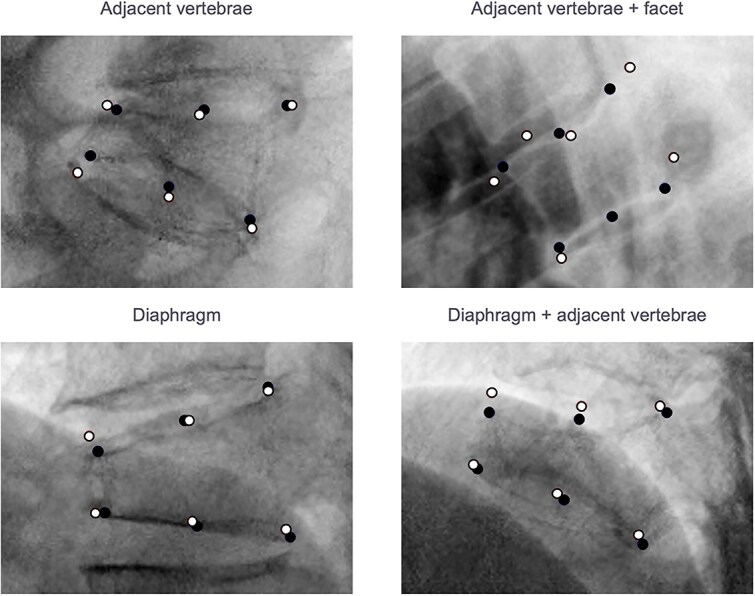
Examples of vertebral bodies showing discrepancies of ≥0.2 in vertebral body height ratios between the algorithm and the training data creator. The black dots and white circles represent the landmarks set by the training data creator and the algorithm, respectively. Errors included incorrect landmark placement on adjacent vertebral bodies, facets, or the diaphragm, resulting in underestimation of anterior and central height reduction.

Furthermore, height ratio discrepancies of ≥0.2 between the algorithm and the reference annotations were observed in eight vertebral bodies (0.8% of the total). These discrepancies were due to the algorithm mistakenly annotating adjacent vertebral bodies, vertebral arches, diaphragms, or posterior airway walls (Figure 4).

### Verification of the second-stage AI using the observer test dataset

**Figure 5:**
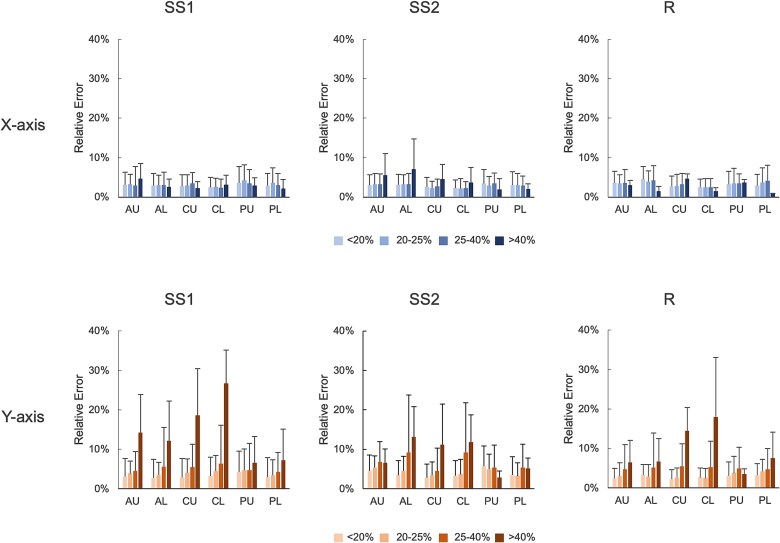
Landmark errors between the algorithm and external evaluators, categorized by the degree of collapse as calculated by the external evaluators. Vertebral bodies with more than 25% collapse showed increased Y-axis errors, particularly at anterior-upper (AU), anterior-lower (AL), central-upper (CU), and central-lower (CL) landmarks. Abbreviations: PL, posterior-lower; PU, posterior-upper.

Landmark errors, evaluated against 3 external evaluators were as follows: for SS1, the X-axis error was 3.0% and the Y-axis error was 3.4%; for SS2, 2.9% and 4.0%; and for *R*, 3.3% and 2.9%. Thoracic images exhibited consistently greater errors across all evaluators. Vertebral bodies with >25% collapse showed increased Y-axis error, particularly in central-upper, central-lower, anterior-upper, and anterior-lower landmarks (Figure 5).

**Figure 6:**
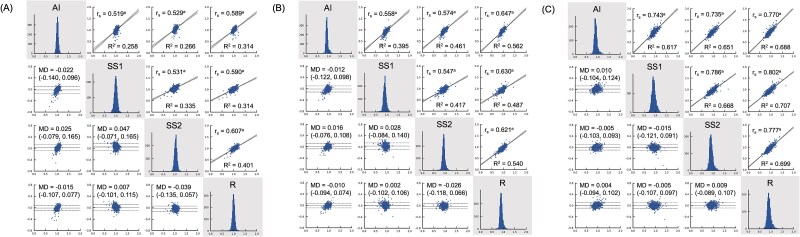
Distributions (histograms with grey backgrounds), correlation (scatter plots on the upper right-hand side, with *r_s_*, *R*,^2^ and regression lines with 95% CI), and agreements (Bland–Altman plots on the lower left-hand side, with MD and LOA) for *C/A* (A), *C/P* (B), and *A/P* (C) ratios calculated by the algorithm and the external evaluators in the observer test dataset. Correlation analyses indicate that *r_s_* values between the algorithm and each external evaluator are comparable with those among the evaluators themselves for each ratio, with values decreasing in the order of *A/P* > *C/P* > *C/A*. Bland–Altman analysis revealed a statistically significant moderate positive trend for the *C/A* ratio between the algorithm and SS1 (slope = 0.696, *p* < .0001, *R*^2^ = 0.311). Mean differences between the algorithm and external evaluators were similar to those observed between the evaluators, with LOA falling within comparable ranges. *n* = 1753. ^a^*p* < 0.0001.Abbreviations: LOA, limit of agreement; MD, mean difference; R, radiologist; *R*^2^, *R*-squared; *r_s_*, Spearman’s correlation coefficient; SS1, spine surgeon 1; SS2, spine surgeon 2.

Figure 6 illustrates the distribution of vertebral body height ratios calculated by the algorithm and the evaluators, along with their correlation and agreement. Correlation analyses showed that *r_s_* values between the algorithm and each external evaluator were comparable with those among the evaluators themselves, with the *r_s_* values decreasing in the order of *A/P*, *C/P*, and *C/A*. Bland–Altman analysis indicated a statistically significant and moderate positive trend in the *C/A* ratio between the algorithm and SS1 (slope = 0.696, *p* < .0001, *R*^2^ = 0.311). A weaker positive trend was observed for the *C/A* ratio (*R*^2^ = 0.195) and *C/P* ratio between the algorithm and SS2 (*R*^2^ = 0.163). The mean differences and LOA between the algorithm and external evaluators were comparable with those observed among the evaluators themselves.

Discrepancies of ≥0.2 in height ratios occurred in 2.4% of cases, with annotation errors arising under condition similar to those identified in the test dataset.

### Measurement times of the proposed algorithm

The proposed algorithm successfully analyzed thoracic and LS images within 10 s per image. While thoracic spine images required slightly longer processing times than LS images, all measurements were completed within the 10-s threshold.

## Discussion

In this study, we developed an AI-based algorithm for QM of the vertebral body. This research served as an exploratory investigation to evaluate the initial performance of the algorithm rather than to meet predefined targets. The algorithm analyzed lateral radiographs of the thoracic and LS, automatically detected 6 key landmarks on the vertebral body, and calculated vertebral body height ratios. The results demonstrated reliable detection of vertebral bodies and efficient processing times, underscoring the algorithm’s potential for clinical applications.

The evaluation results indicated that the algorithm’s correlation and agreement with external evaluators were comparable with the variability observed among the evaluators themselves, suggesting satisfactory performance. However, certain challenges were identified. The landmark error analysis revealed increased variability at anterior-upper, anterior-lower, central-upper, and central-lower landmarks, particularly as vertebral collapse progressed. Discrepancies of ≥0.2 in height ratios were observed in some cases, likely contributing to an underestimation of anterior and central height collapse. These findings are consistent with the lower correlations observed in the C/*A* and *C/P* ratios, especially in cases involving central height. The Bland–Altman analysis of both the test dataset and the observer test dataset revealed positive linear trends in the *C/A* ratio and, to a lesser extent, in the *C/P* ratio, indicating potential systematic bias. This bias may result from the algorithm’s tendency to underestimate anterior and central height collapse. For example, in biconcave or crush deformities where the *C/A* ratio decreases, underestimation of central height could lead to higher *C/A* ratios compared with external evaluators, causing deviations to the lower left quadrant of the Bland–Altman plot. Conversely, in wedge deformities where the *C/A* ratio increases, underestimation of anterior height could result in lower *C/A* ratios, shifting points to the upper right quadrant. A similar pattern was observed for the *C/P* ratio: underestimation of central height in wedge or biconcave deformities led to higher *C/P* ratios and deviations to the lower left quadrant, while crush deformities caused deviations to the upper right quadrant. These results suggest that, particularly in vertebrae with significant collapse, the algorithm’s underestimation of anterior and central heights warrants careful interpretation. In such cases, manual adjustments may be necessary to ensure accurate measurements.

Compared with manual QM, which can take over 10 min per radiograph, the proposed algorithm dramatically reduces analysis time to under 10 s per image. With user verification, most images are processed in less than 1 min. Existing semi-automatic systems that utilize statistical decomposition methods^30–32^ typically require manual vertebral detection and approximately 7 min per case. In contrast, our algorithm performs fully automatic detection of vertebral bodies. Moreover, while Suri et al.’s system^22^ can analyze the whole spine using CT and MRI, its radiography-based analysis is limited to T10-L5. Our algorithm enables comprehensive evaluation of both the thoracic (T4 and below) and LS using widely accessible radiographs, offering a significant advantage for routine clinical use. The short analysis time also makes it highly suitable for large-scale osteoporosis screening and clinical research applications.

This study explored QM while emphasizing the need for caution when using it to diagnose vertebral fractures. Recent research suggests that qualitative evaluations focusing on endplate damage may be more effective for diagnosing new vertebral fractures and assessing the risk of subsequent fracture. Such evaluations have demonstrated significant associations with low bone mineral density^16^^,^^33^ and the occurrence of both vertebral and non-vertebral osteoporotic fractures.^16^^,^^34^ In contrast, QM may increase false-positive rate by misidentifying non-fragility deformities, such as Schmorl’s nodes and Scherermann’s disease, as fractures.^35^^,^^36^ While QM does not directly assess endplate damage, it may indirectly reflect this through measurements of central vertebral body height.^15^ The proposed algorithm, which allows for manual landmark adjustment and post-analysis evaluation of endplate damage, has the potential to enhance the diagnostic accuracy of vertebral fractures.^37^

This study, however, has several limitations. First, there is a “ground truth” challenge in accurately establishing landmarks using only radiographs. Although integrating CT or MRI data could improve accuracy, this approach was not implemented in this study. The training data were developed based on the landmark creation method proposed by Genant et al.^15^ and verified by external experts to address subjectivity and accuracy concerns. Nevertheless, ambiguities in landmarking using lateral radiographs alone were unavoidable. Future studies could incorporate this algorithm into CT imaging, which provides higher resolution and 3D views. This could enhance landmark accuracy, enable precise assessments of vertebral deformities, and facilitate the evaluation of surrounding structures, such as musculature, to gain deeper insights into factors contributing to vertebral fractures. Second, there was variability in the degree of vertebral body collapse represented in the training data. Ideally, the training dataset would include a balanced distribution of vertebrae with varying levels of collapse. However, over 90% of the training data consisted of vertebrae with less than 20% collapse. Consequently, the algorithm’s agreement with the training data creator diminished for the vertebrae with greater degree of collapse in the test dataset. This imbalance resulted from the random selection of clinical images over a specific period. Targeted training with a greater emphasis on vertebrae exhibiting significant collapse could potentially improve the algorithm’s performance in such cases. Finally, there is concern regarding the algorithm’s ability to accurately process cases of scoliosis. Scoliosis cases with a Cobb angle of ≥15° were excluded from the training, test, and observer test datasets. Therefore, the algorithm’s performance in such cases remains unassessed.

In conclusion, we developed an AI-based algorithm for the QM of vertebral bodies. The algorithm analyzed lateral radiographs of the thoracic and LS, automatically detected 6 landmarks on the vertebral body, and calculated the vertebral body height ratios. The results demonstrated sufficient correlation and agreement with the external evaluators, confirming the algorithm’s reliability. However, the evaluation results indicate the need for caution, as the algorithm may underestimate anterior and central height reduction, particularly in cases of moderate to severe vertebral collapse. The algorithm achieved high sensitivity and precision in vertebral body detection, with a processing time of less than 10 s per image. This efficiency positions the algorithm as a valuable tool for improving the evaluation of vertebral body deformities, aiding in the diagnosis of vertebral fractures and osteoporosis, and supporting timely and appropriate clinical treatment.

## Data Availability

The data supporting the findings of this study are available from the corresponding author upon reasonable request.
